# Aerial insulator defect detection method based on CWSP-YOLO

**DOI:** 10.1038/s41598-026-40782-2

**Published:** 2026-04-17

**Authors:** Zhenjun Du, Yixin Geng, Hucheng Wang, Hengchang Zhang, Yanjun Hu

**Affiliations:** State Grid Gansu Provincial Electric Power Company Tianshui Power Supply Company, Tianshui, 741000 China

**Keywords:** Engineering, Mathematics and computing

## Abstract

With the rapid evolution of UAV technology, intelligent power equipment inspection via aerial imagery is critical. To address traditional insulator defect detection bottlenecks (high false/missed detection, insufficient multimodal fusion), this paper proposes an improved YOLOv11-based model integrated with multimodal data, featuring cross-modal collaboration, wavelet-optimized C3k2, channel attention, and PIoU v2-based dynamic gradient optimization. Experiments on a self-built dataset show it achieves 84.77% mean average precision, 94.53% accuracy, 82.38% recall, with 24 FPS meeting real-time requirements, offering reliable support for UAV power inspection.

## Introduction

With the rapid development of unmanned aerial vehicle (UAV) technologyand^1–3^ and image recognition technology^4–6^, and the growing demand for intelligent inspection of power systems, defect detection of power equipment based on aerial images has become one of the key technologies to ensure the safe and stable operation of power grids. As a crucial component in transmission lines, surface defects of insulators (such as cracks, breakages, and contamination) that are not detected in a timely manner can easily lead to serious accidents like flashovers and power outages^7–9^. Traditional automatic detection methods based on visible light suffer from high false detection and missed detection rates in scenarios with complex illumination, small targets, and occlusions^10–12^. In recent years, the introduction of multimodal sensing technologies (e.g., infrared imaging and LiDAR) has provided complementary information for defect detection; however, effectively fusing multi-source data and achieving high-precision real-time detection remain key challenges to be addressed^13^.

Currently, scholars have conducted extensive research in the field of insulator defect detection. Reference^14^ improved the YOLOv11n framework by embedding the C3k2-SG module in the backbone network, integrating a diffused pyramid network to realize multi-scale feature diffusion and fusion, and adopting a lightweight FPSConv operator to balance computational efficiency and detail capture. Reference^15^ introduced a dual-layer routing attention mechanism into the backbone network to enhance global feature representation and suppress noise interference, optimized cross-scale feature fusion via a weighted bidirectional feature pyramid, and reconstructed the Neck network to eliminate redundant nodes. Reference^16^ enhanced the backbone network’s ability to capture detailed features of small targets by introducing a cross-layer connection structure and a tiny target detection layer, and optimized multi-scale localization perception with a lightweight dynamic detection head (GSConv), significantly alleviating missed and false detections of dense small targets in UAV aerial scenarios. Reference^17^ proposed the lightweight network FNM-Net, which reduces feature redundancy by replacing the backbone network with Faster-Net, optimizes cross-layer feature fusion using a focus modulation module and a spatial information integration module, designs multi-fine-grained detection heads to adapt to target scale changes, and employs the LAMP pruning strategy to compress the model parameter count.

Although existing methods have improved the YOLO algorithm to a certain extent, they generally still have the following limitations: single-modal data struggles to cover complex working conditions (e.g., strong reflections, haze).

Reference^18^ proposes the ME-YOLO algorithm, which introduces the MGDB module (originally designed for high-resolution image restoration tasks) to enhance the detection accuracy for different modal images. However, the model can only process one type of modal image at a time, failing to achieve modal fusion and resulting in insufficient utilization of complementary information. Reference^19^ presents the BPP-YOLO model, which introduces a fusion module in the middle stage of feature extraction to perform weighted fusion of features at different scales, improving the detection capability in dark environments. Nevertheless, fusing at the feature level leads to the loss of some original information. Reference^20^ proposes the ACFI-YOLO algorithm, which uses a Transformer encoder to conduct cross-layer information interaction on feature vectors, thereby enhancing the complementarity between shallow and deep information. However, it also cannot process multiple types of modal information simultaneously. Meanwhile, these papers also suffer from insufficient balance between lightweight design and detection accuracy, making it difficult to meet the real-time requirements of UAV edge computing.

To address the above issues, this paper proposes a multi-modal aerial insulator defect detection method (CWSP-YOLO) based on an improved YOLOv11 architecture. First, a cross-modal collaborative perception mechanism is constructed, and a mid-term fusion strategy is adopted to achieve in-depth interaction at the feature level, enhancing the saliency representation of defect regions through cross-modal feature complementarity. Second, a channel attention module is embedded in the backbone network to establish a channel feature response screening mechanism, strengthening the feature expression capability of key channels. Furthermore, the PIoU v2 loss function is introduced, and a dynamic adjustment mechanism of gradient weights is used to optimize the gradient propagation efficiency of medium-quality anchor boxes.

## Background of YOLOv11

YOLOv11 is an object detection algorithm released by Ultralytics on September 30, 2024^21,22^. Compared with previous YOLO series models, YOLOv11 has achieved significant improvements in both accuracy and speed.

The YOLOv11 detection model consists of three components: the backbone network, the neck network, and the head network.

### Backbone network

The backbone network of YOLOv11 employs an optimized CSPDarknet53 framework, which generates multi-resolution feature maps through five stages of scale compression processes. This backbone network integrates the novel Spatial Pyramid Fast Pooling (SPFF) component, which unifies feature sizes via multi-scale pooling operations to effectively enhance the richness of feature representation. Additionally, it incorporates the Pyramid Slicing Attention (C2PSA) mechanism, which strengthens the model’s ability to capture key features.

### Neck network

The neck network of YOLOv11 adopts the PAN-FPN architecture. Through a bottom-up feature transmission path, it achieves the organic integration of shallow-layer localization information and deep-layer semantic features, which effectively addresses the localization accuracy limitation of the traditional FPN.

### Head network

The detection head adopts a dynamic decoupled design, where the feature channels for classification and regression tasks are completely separated. It generates task-specific weight matrices through lightweight dynamic convolution kernels. Additionally, Probabilistic Non-Maximum Suppression (Probabilistic NMS) is introduced, which fuses the probability distributions of confidence scores and IoU (Intersection over Union) to optimize the detection ranking strategy for dense targets.

## CWSP-YOLO

### Overall block diagram of the improved YOLO

**Fig. 1:**
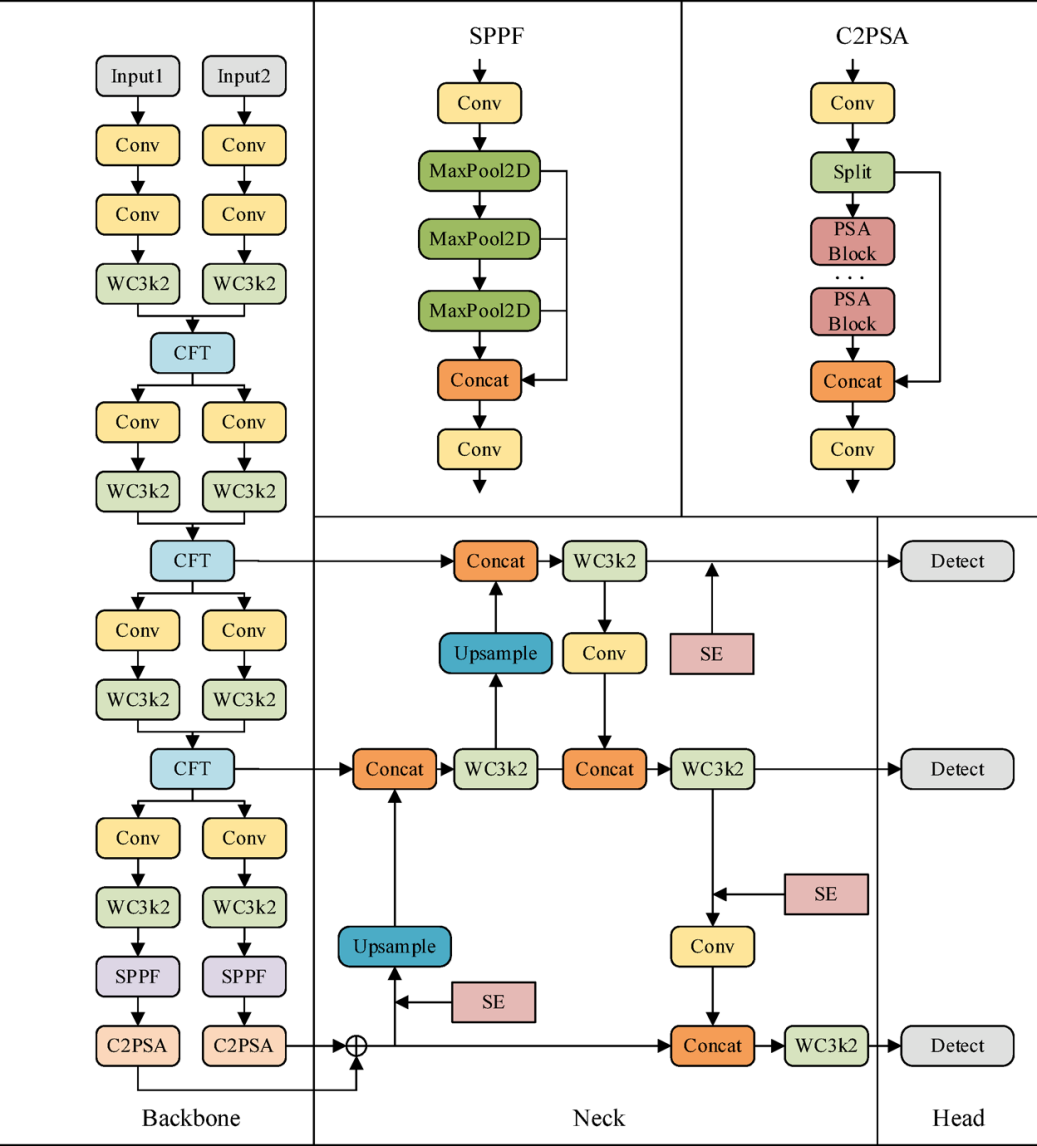
CWSP-YOLO network framework diagram.

The improved model proposed in this paper is designed for the task of insulator defect detection in aerial photography scenarios, aiming to fuse the image features of two modalities—visible light and infrared images—and give full play to their respective advantages. Based on this objective, enhanced designs are implemented for the backbone network, neck network, and prediction network of the YOLO model. The overall block diagram of the improved model is shown in Fig. 1.

Among them, the backbone network is a dual-stream backbone network improved based on the Cross-Modality Fusion Transformer (CFT); the Neck is an enhanced backbone network improved based on the attention module (squeeze and excitation, SE); and the Head is a dynamically adjusted prediction network after the introduction of the PIoU v2 (Powerful-IoU v2) loss function.

### Cross-modality fusion transformer

**Fig. 2:**
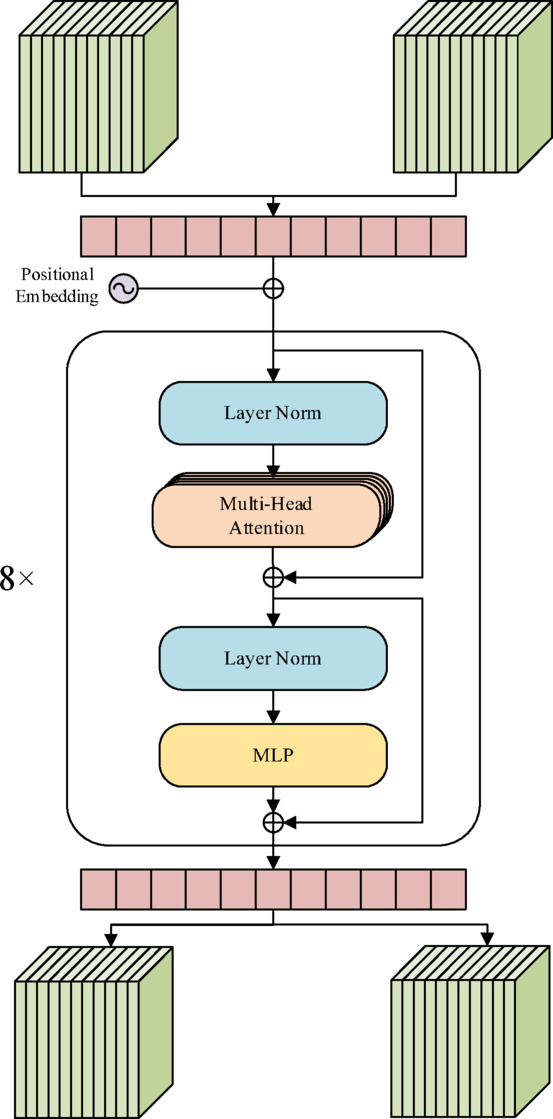
CFT module flowchart.

The Cross-Modality Fusion Transformer (CFT) is a cross-modal feature fusion module based on the Transformer architecture, which consists of three core operations: modal feature concatenation, self-attention interaction, and residual enhancement^23^. By globally modeling the long-range dependencies between RGB and thermal imaging modalities, it can effectively fuse complementary information and suppress redundant features. The structure of the CFT module is shown in Fig. 2.

The module flattens and concatenates the RGB and thermal imaging feature maps input to the CFT module into a sequence, and embeds learnable positional encoding. Then, it calculates the cross-modal and intra-modal attention weight matrices via the multi-head self-attention mechanism, and learns the global interaction patterns between features. Finally, it processes the fused features output by the Transformer through downsampling and upsampling, adds them to the original features in a residual form, and dynamically enhances the saliency representation of target regions.


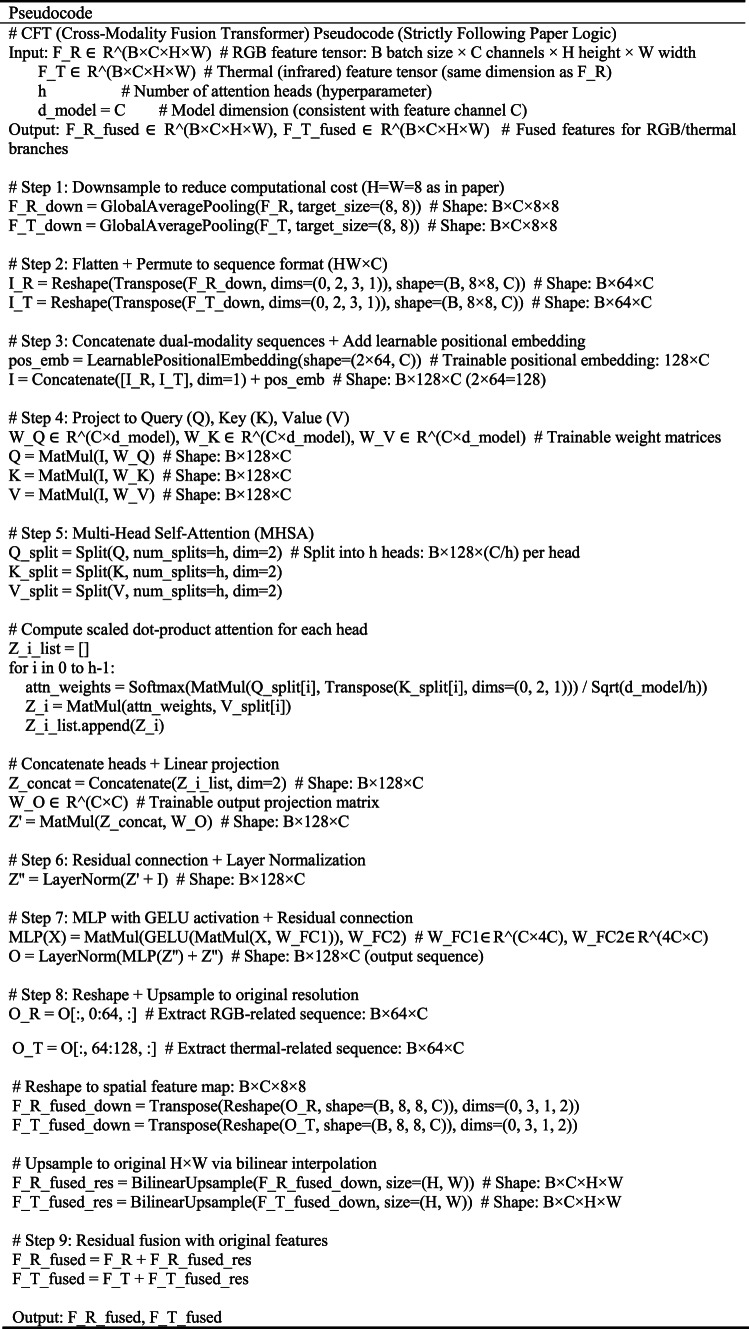


The CFT module performs fusion operations on RGB photos and infrared photos in the original image dimension without losing any original information, and utilizes the powerful capabilities of the Transformer architecture to extract relevant information. During training and usage, the UAV captures one RGB photo and one infrared photo consecutively at the same location, forming one sample set. Even if there are vertical or horizontal positional differences in the spatial arrangement of the two cameras, the imaging perspective difference between the two photos is small. They can be used in the algorithm after only simple distortion correction, without the need for complex registration work. For the specific information of the UAV and cameras, refer to the subsequent experimental chapter.

### Wavelet transform convolutional module

**Fig. 3:**
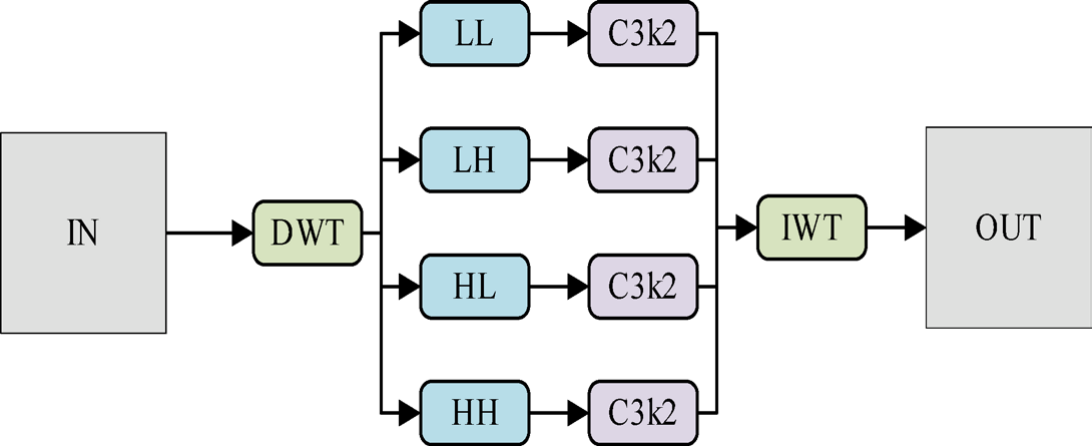
WC3k2 module flowchart.

The imaging environments of transmission lines in the field are usually relatively complex. Additionally, different shooting angles of UAVs result in uncertain imaging scales of the detected targets, and different insulator damage types lead to irregular damage features. To expand the receptive field for effective extraction of image information while avoiding excessive increase in network parameters, the C3k2 module is improved by adopting Haar wavelet transform. Firstly, the input data is processed via wavelet transform and divided into frequency-specific information sub-bands. Then, C3k2 convolutional modules are connected in different frequency channels. After information extraction, through inverse wavelet transform and information concatenation, the key features of the original information are effectively retained. The structure of WC3k2 is shown in Fig. 3.

After the original information passes through the wavelet transform module, low-frequency sub-bands and high-frequency sub-bands are obtained. The low-frequency sub-bands carry global information features, while the high-frequency sub-bands carry detailed information features. The information processed by convolution can effectively suppress background noise and improve the robust extraction capability under noise interference. Meanwhile, the decomposition and analysis of high and low frequencies fully retain various information features, which can significantly enhance the adaptability to complex environments and targets captured from multiple angles.


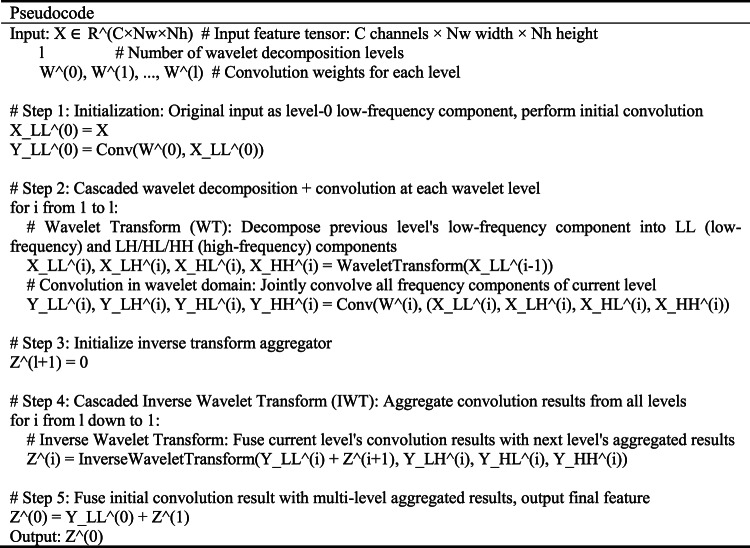


### Squeeze-and-excitation attention module

**Fig. 4:**
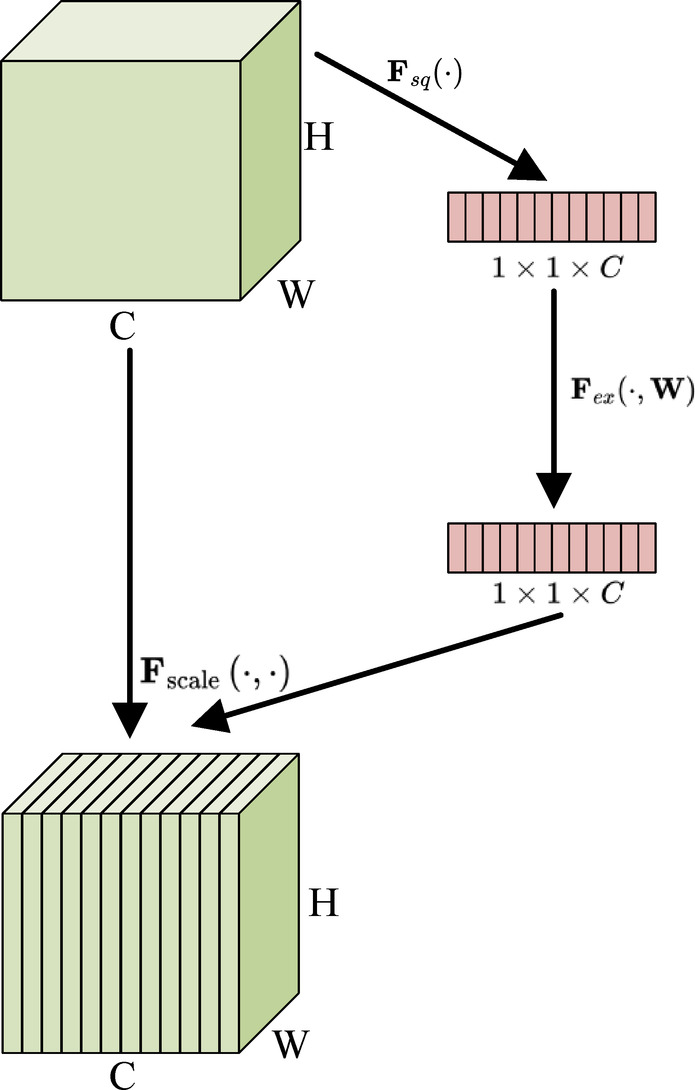
SE attention module flowchart.

The Squeeze-and-Excitation (SE) attention mechanism, as a classic model in the field of channel attention, consists of three key stages in its core process: global information squeezing, channel weight self-learning, and feature recalibration. By dynamically capturing non-linear correlations between channels, this module achieves enhanced focus on key feature channels and suppression/attenuation of non-essential features. Its workflow is shown in Fig. 4^24^.

First, a spatial squeezing process is applied to the input feature tensor, and contextual information across spatial dimensions is aggregated via Global Average Pooling (GAP). Subsequently, a bottleneck-style fully connected layer structure containing dimension reduction and dimension elevation operations is constructed. Finally, the channel weights are subjected to non-linear normalization processing through the Sigmoid function, and the obtained importance coefficients are weighted and fused with the original input features to realize an adaptive feature enhancement mechanism.


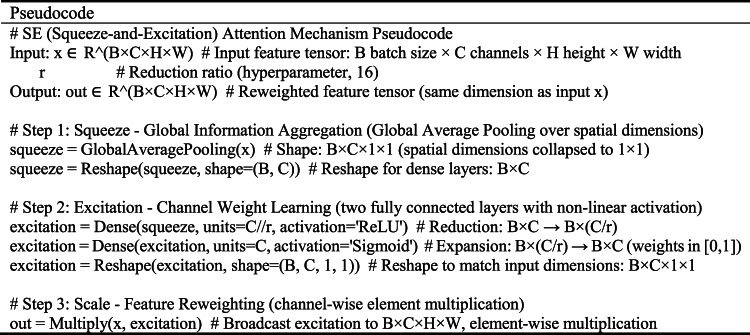


### Loss function optimization

The original YOLOv11 framework adopts the CIoU loss function, which is improved based on the IoU (Intersection over Union) algorithm. It is characterized by simultaneously integrating the Intersection over Union (IoU) between predicted boxes and ground truth boxes, the offset of the center points between them, and the consistency of their aspect ratios. However, since the penalty term design depends on the size of the external bounding box, it leads to meaningless expansion of anchor boxes during the regression process and slows down the training speed. More importantly, when the aspect ratio difference between predicted boxes and ground truth boxes is extremely large, the calculation may cause training failure due to gradient explosion or vanishing.

To address these issues, we adopt the improved PIoU v2 loss function. By introducing a target size-adaptive penalty factor, adopting a non-monotonic attention mechanism, and dynamically adjusting the gradient of the penalty term, it further improves the convergence speed and detection accuracy^25^. Details are specifically shown in Eqs. (1–6):

1 $$L_{{{\mathrm{PIoU}}\_{\mathrm{v2}}}} = u(\lambda q) \cdot \left( {L_{{{\mathrm{IoU}}}} + f(P)} \right)$$

2 $$u(\lambda q) = 3 \cdot (\lambda q) \cdot e^{{ - (\lambda q)^{2} }}$$

3 $$q = e^{{ - P}}$$

4 $$L_{{{\mathrm{IoU}}}} = 1 - \frac{I}{U}$$

5 $$f(P) = 1 - e^{{ - P^{2} }}$$

6 $$P = \frac{{\frac{{d_{{w1}} }}{{w_{{gt}} }} + \frac{{d_{{w2}} }}{{w_{{gt}} }} + \frac{{d_{{h1}} }}{{h_{{gt}} }} + \frac{{d_{{h2}} }}{{h_{{gt}} }}}}{4}$$

Where: $L$ denotes the derived PIoU v2 loss function; $u$ is the attention function, which optimizes the model’s regression process for anchor boxes through non-monotonic gradient weight allocation; $f$ represents the gradient adjustment function, which optimizes the bounding box regression process by dynamically adjusting the gradient magnitude; $P$ denotes the target size-adaptive penalty factor, which can be dynamically adjusted according to the size of target boxes to suppress the expansion of anchor boxes; $\lambda$ is the hyperparameter controlling the behavior of the attention function, in this paper, we set $\lambda$ to 1.3; $q$ measures the quality of the anchor box; $I$ represents the intersection of the predicted box and the target box, $U$ represents their union; ${d_{w1}}$, ${d_{w2}}$, ${d_{h1}}$, and ${d_{h2}}$ are the absolute values of the distance between the corresponding adges of the predicted box ang the target box, ${w_{gt}}$ and ${h_{gt}}$ are the width and height of the target box.

## Results

The hardware of the experimental platform in this study is built with an Intel Core i7-13700 processor and an NVIDIA GeForce RTX 4090 graphics card. The software system is constructed based on the Ubuntu operating system, and the development toolchain includes the Python programming language, the PyTorch deep learning framework, and the PyCharm integrated development environment (IDE).

### Evaluation metrics

This study selects four items as model evaluation metrics, including mean average precision (mAP), precision (P), recall (R), and frame rate. The specific calculations are shown in Eqs. (7–10):

7 $$P = \frac{{TP}}{{TP + FP}} \times 100\%$$

8 $$R = \frac{{TP}}{{TP + FN}} \times 100\%$$

9 $$AP = \int_{0}^{1} P (r){\mathrm{d}}r$$

10 $$AP = \int_{0}^{1} P (r){\mathrm{d}}r$$

### Training process

**Fig. 5:**
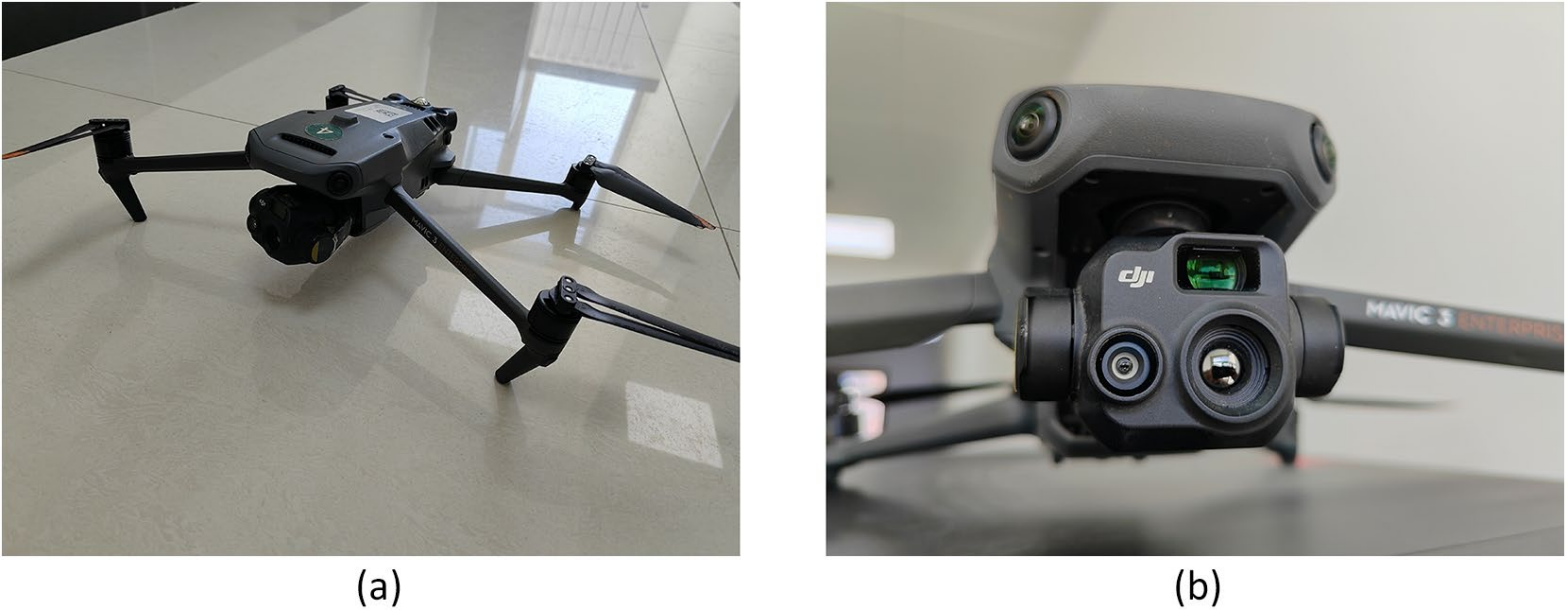
UAVs and cameras.

The dataset used in this paper consists of RGB and thermal infrared images collected by UAVs in practical work, which is shown in Fig. 5. The UAV camera is equipped with a multimodal camera, which can capture RGB and thermal images simultaneously. The RGB images have a resolution of 3840*2160, and the thermal images have a resolution of 640*512. The UAV has a maximum FOV of 82 degrees. Its effective shooting distance ranges from 1 m to infinity, and the UAV usually operates within a safe range of 5–10 m from the transmission lines and transmission towers. It can operate in Level 7 wind conditions. The operating temperature is − 30 to 50 °C. The RGB camera can work under all non-backlit conditions.

**Fig. 6:**
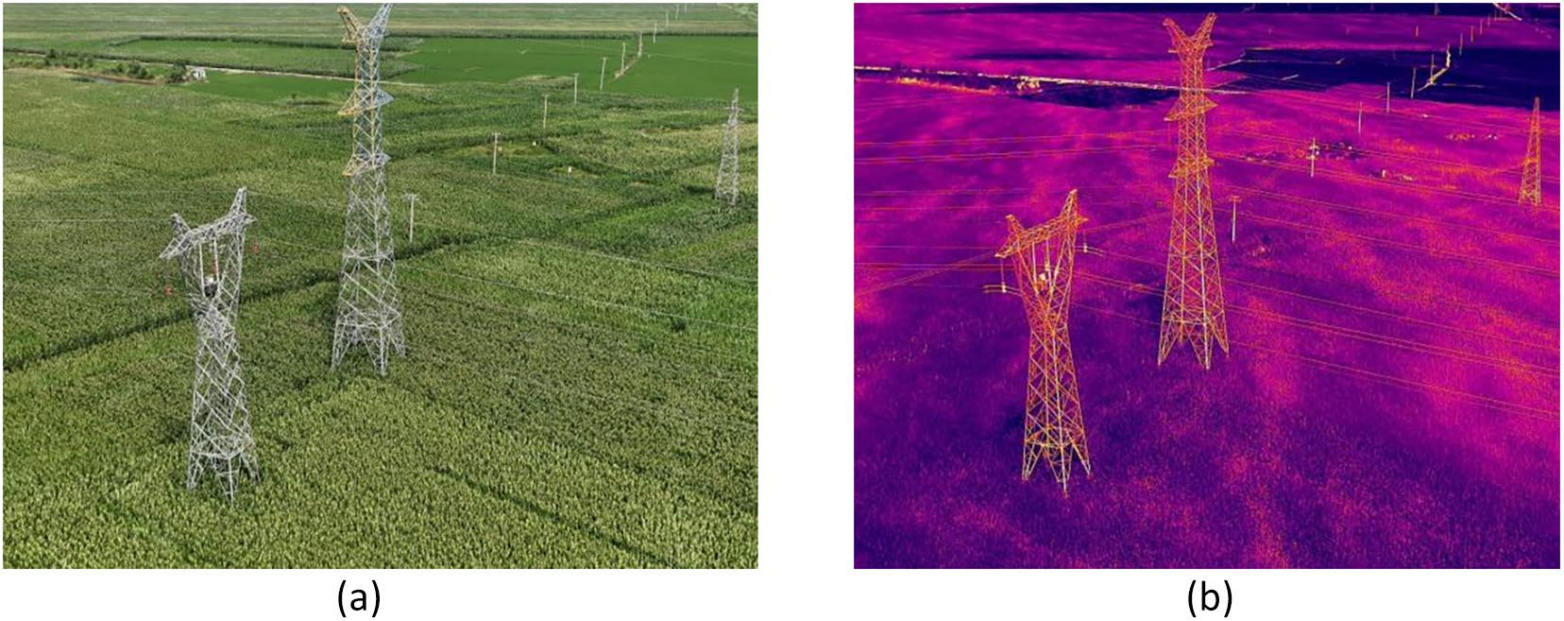
Example image in dataset.

To collect insulator images for algorithm training, during daily inspections, operation personnel will operate UAVs to sequentially use RGB cameras and infrared cameras for shooting at the same location. The two consecutively taken photos will be automatically paired by the backend into a set of photos. In the later stage, operation personnel will identify the status of insulators in the photos by group, use the LabelImg to mark the insulators in the photos as well as possible overheating and fragmentation issues, thus forming a training dataset. The example images are shown in Fig. 6. The dataset has 1143 images. The images are divided into 4 categories: normal pin insulators, normal disc insulators, defective pin insulators, and defective disc insulators, including 224 valid defective samples (Overheating, self-explosion, breakage, et al.). Considering the small scale of defective samples, diverse samples are generated through data augmentation strategies using rotation (± 30°), random cropping (scaling ratio 0.8–1.2), brightness adjustment (± 20%), and adding Gaussian noise (σ = 0.05). The total number of samples is finally expanded to 5000, and the new dataset is divided into training set, validation set, and test set at an 8:1:1 ratio in various different ways using the K-Fold Cross-Validation method, and repeated the training process multiple times to ensure the generalization ability of the model.

**Fig. 7:**
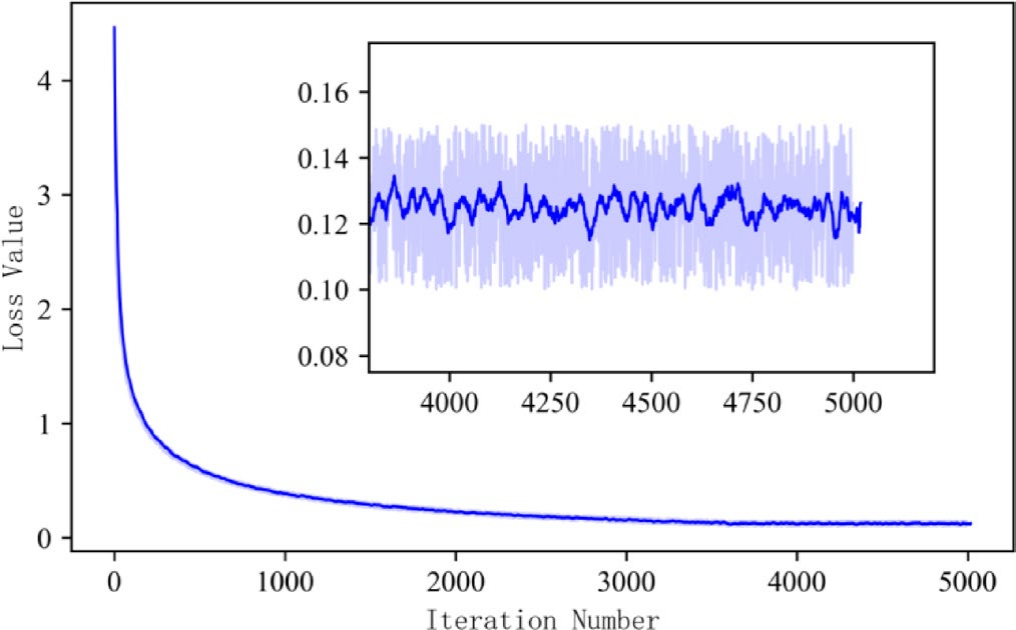
Loss curves of CWSP-YOLO model.

In the training process of the CWSP-YOLO model, input images are uniformly cropped and resized to a resolution of 640 × 640 pixels, and network parameters are initialized with COCO pre-trained weights. The training adopts basic configurations including a batch size of 64, a weight decay coefficient of 0.0005, and a momentum factor of 0.9, and completes optimization through 5000 iterations. To meet the requirement of training stability, a dynamic learning rate adjustment strategy is implemented: the base learning rate of 0.01 is maintained for the first 3000 iterations to accelerate convergence; it is gradually decayed to 0.002 from iteration 3000 to 4500 to refine feature learning; finally, a fine-tuning learning rate of 0.0002 is used in the last 500 iterations to enhance model robustness. In this paper, the convolution kernel of the convolutional layer is set to 3, and the stride is set to 2. The reduction ratio of SE is set to 16. The entire training process takes 6 h on a single RTX 4090 platform, and the loss function eventually converges to a stable threshold of 0.23. The loss variation trend during training is shown in the visualized curve in Fig. 7.

### Comparative experiment

In this paper, we incorporate CFT into the model to enable detection using information from both visible and infrared modalities simultaneously. To verify the effectiveness of dual-modal detection compared with single-modal detection, we list several comparative experiments.

**Fig. 8:**
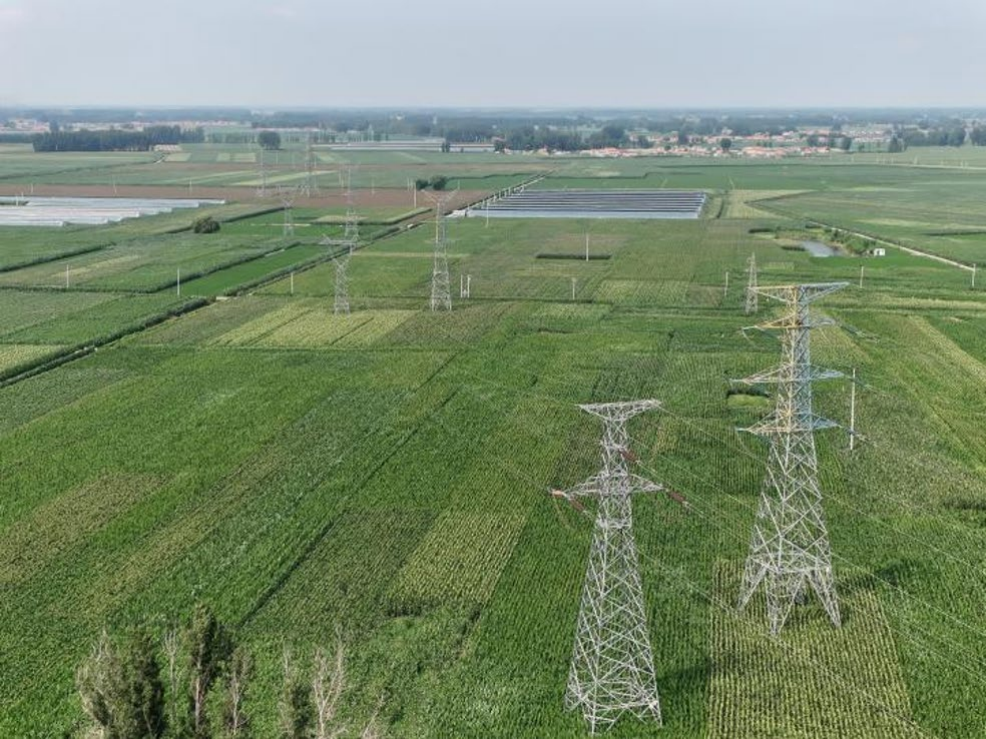
Images captured during daily inspections.

**Fig. 9:**
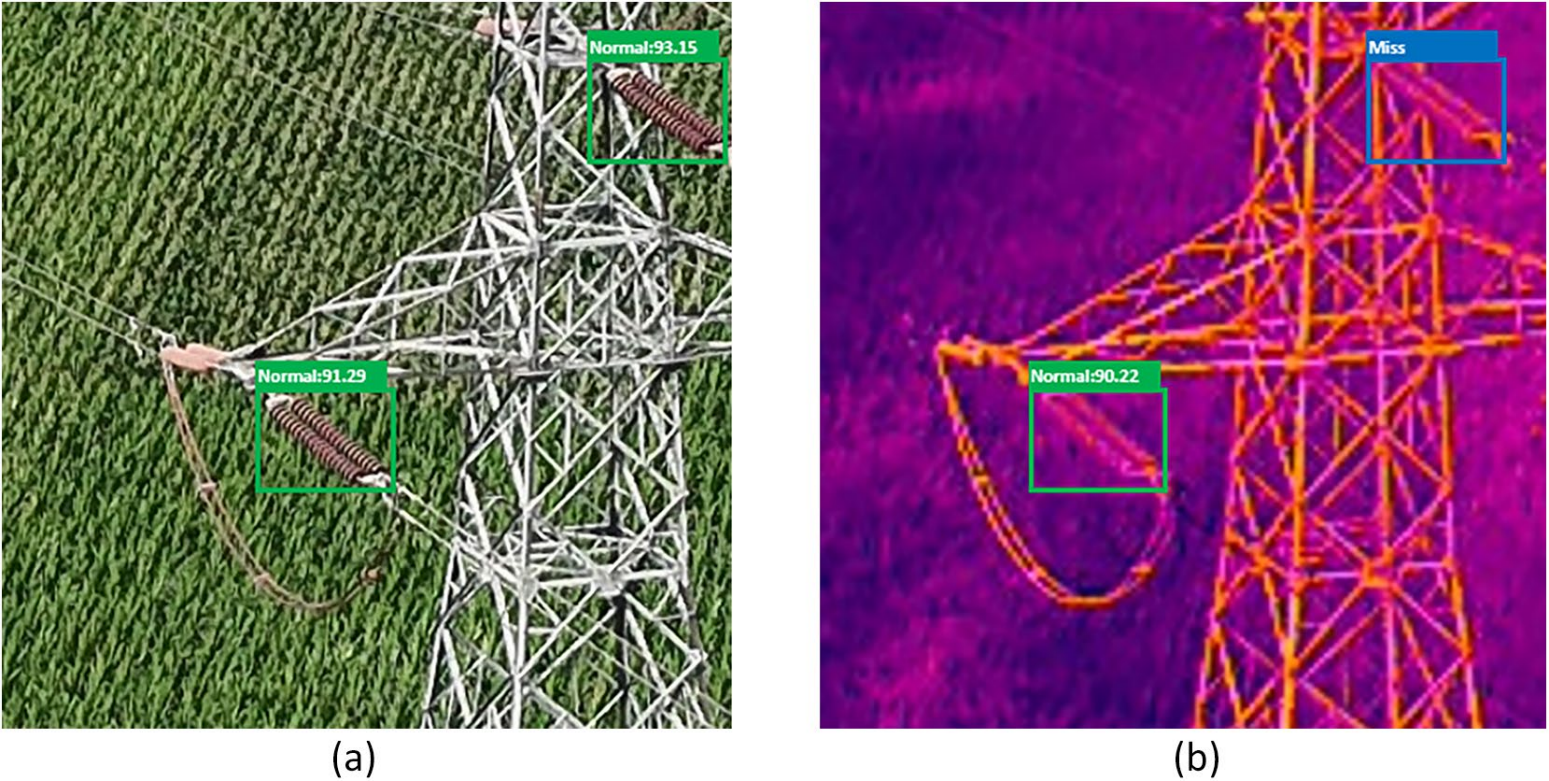
Visual comparison of insulator defect detection results.

In many scenarios, to ensure the safety of the power grid and operations, UAVs are generally not allowed to approach transmission lines for close-range shooting (see Fig. 8). At this time, due to the low resolution of infrared cameras, it is difficult for them to perform high-magnification zoom on images like visible light cameras, thus posing challenges to fault identification. As shown in Fig. 9, in the visible light view, the edges of the insulator string are clear and the insulator discs can still be distinguished, while in the infrared view, the insulator discs cannot be accurately identified due to low resolution.

**Fig. 10:**
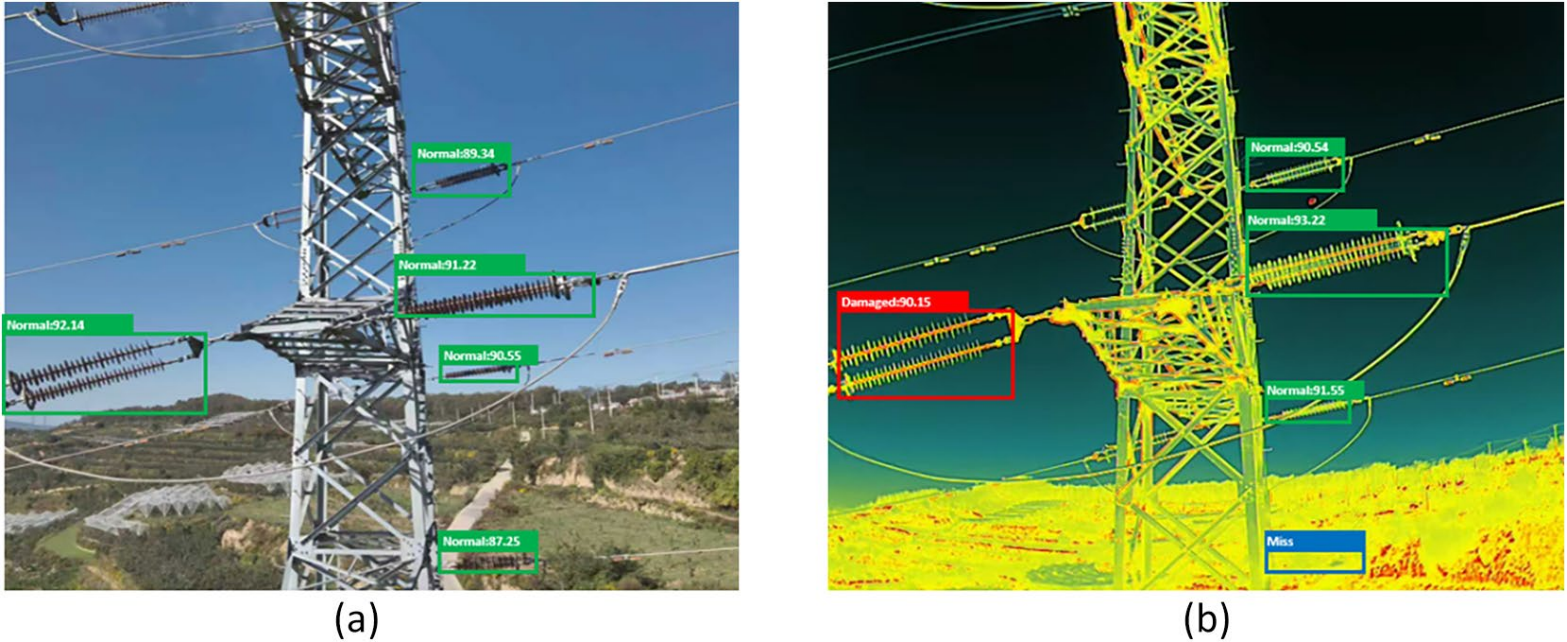
Visual comparison of insulator defect detection results.

However, for issues such as abnormal heating of insulators, infrared cameras must be used for detection (see Fig. 10, clearly visible in the figure is the heating of the insulator string on the left).

To quantitatively demonstrate the advantages of the multimodal model, we designed comparative experiments to compare the differences between the single visible light model, the single infrared model, and the multimodal model combining visible light and infrared. The results are shown in the attached Table 1. It can be observed that compared with the single-modal models, the multimodal model, while the processing speed has decreased slightly, exhibits significant advantages in recognition accuracy.

**Table 1:** Results of experiments withRGB and IR.

Model.	mAP50	mAP	*P* (%)	*R* (%)	FPS (f/s)
RGB	76.18	55.85	88.54	78.18	28
IR	73.25	51.26	84.28	75.26	29
RGB + IR	84.77	61.15	94.53	82.38	24

**Fig. 11:**
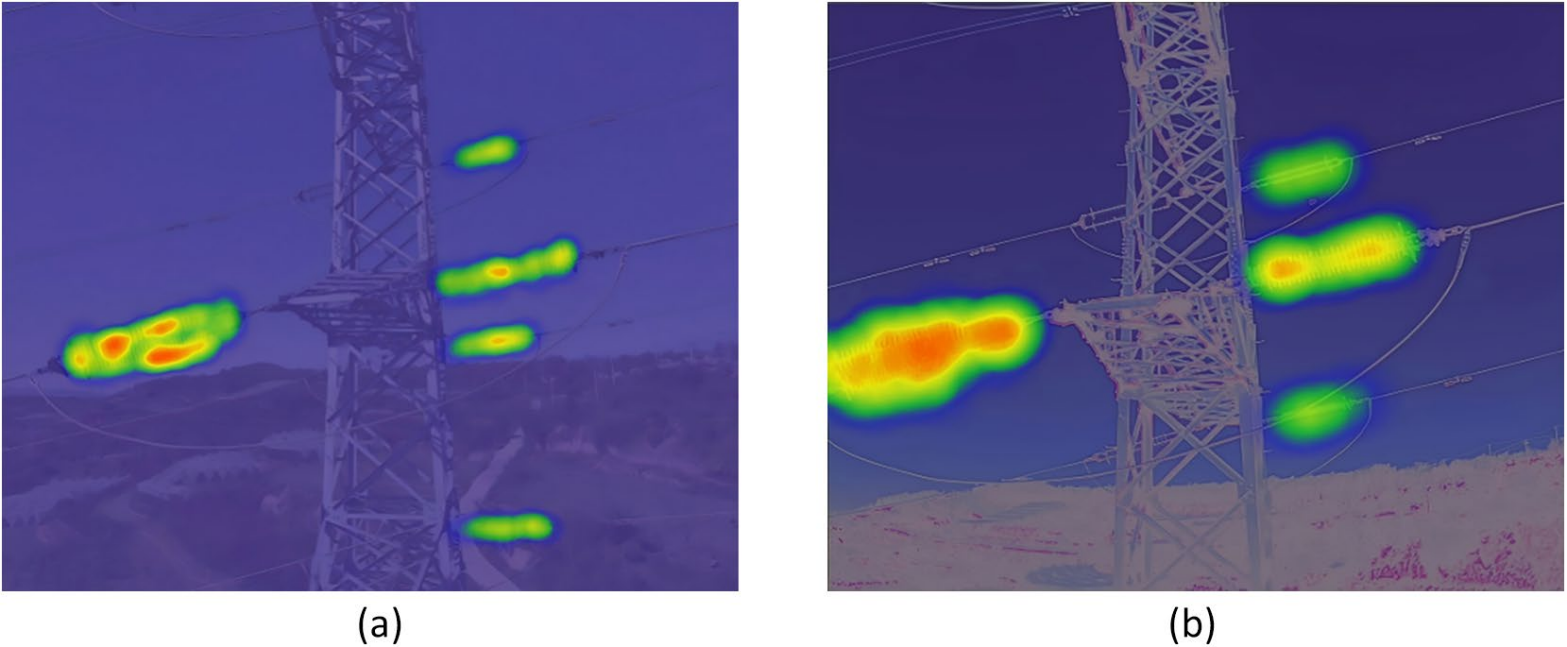
Grad-CAM maps of the model.

Meanwhile, to verify the effectiveness of the CFT multimodal fusion module, we also specifically plotted Grad-CAM maps as shown in the following figure. It can be observed that the model has certain differences in attention distribution across images of different modalities: in the visible light modality, the model’s attention is more comprehensive to avoid missing detections; while in the infrared modality, the model pays greater attention to abnormal heating conditions. This reflects the difference in the model’s attention focus under different modalities, proving that fusing multimodal technology is more conducive to detecting insulator defects (Fig. 11).

### Ablation experiment

To verify the effectiveness of each improved method, this study conducted ablation experiments on the dataset for the following components respectively: the backbone network improved based on CFT, the Wavelet Transform Convolutional Module (WC3k2), the enhanced neck network integrated with the SE attention module (SE), and the improved loss function (PIoU v2). The results are shown in Table 2.

**Table 2:** Results of ablation experiments on the dataset.

No.	CFT	WC3k2	SE	PIoU v2	mAP50	mAP	*P* (%)	*R* (%)	FPS (f/s)
1	×	×	×	×	77.92	54.56	86.22	71.71	22
2	√	×	×	×	82.47	59.88	92.02	81.34	17
3	×	√	×	×	81.56	58.15	91.04	79.23	19
4	×	×	√	×	81.18	59.43	91.57	80.17	20
5	×	×	×	√	79.31	55.12	87.05	76.48	27
6	√	√	×	×	83.12	60.25	92.87	81.85	15
7	√	×	√	×	83.35	60.42	93.15	82.01	16
8	√	×	×	√	82.96	60.13	92.58	81.67	21
9	×	√	√	×	82.84	60.07	92.73	81.72	18
10	×	√	×	√	82.15	59.06	91.86	80.54	23
11	×	×	√	√	81.79	59.68	92.03	80.89	24
12	√	√	√	√	84.77	61.15	94.53	82.38	24

It can be seen from Table 2 that, compared with the traditional YOLOv11 model (Experiment 1), after improving the backbone network based on CFT (Experiment 2), both the Average Precision (AP) and Precision (P) for insulator defects are improved; however, due to the computational overhead caused by module superposition, the target detection speed decreases slightly.

After improving the C3k2 module using Wavelet Transform (Experiment 3), both AP and P for insulator defects are enhanced. Yet, the model’s speed drops somewhat, which is attributed to the computational overhead brought by Wavelet Transform and Inverse Wavelet Transform.

After integrating the SE attention module into the enhanced neck network (Experiment 4), compared with the traditional model, both AP and P for insulator defects are improved, while the model’s speed remains basically unchanged.

After improving the model with the PIoU v2 loss function (Experiment 5), by optimizing training convergence and reducing invalid anchor box regression, the model’s inference efficiency is indirectly improved. Compared with the traditional model, AP, P, and detection speed for insulator defects are all enhanced.

Experiments 6–11 adopt combinations of different improvement measures, all of which have achieved certain improvements compared with the original model, proving that different improvement methods can exert a good synergistic effect.

As can be seen from Experiment 12 in Table 1, by combining the four improvement methods for the YOLOv11 network, compared with the original model, the AP, P, and Recall (R) for insulator defect recognition are increased by approximately 7.79%, 9.63%, and 13.07% respectively. Meanwhile, the model’s detection speed is also improved to a certain extent.

The confusion matrix is shown in the following Table 3.

**Table 3:** Confusion matrix of the experiments.

	Damaged (True)	Normal (True)
Damaged (Predicted)	TP = 173	FP = 10
Normal (Predicted)	FN = 37	TN = 280

**Fig. 12:**
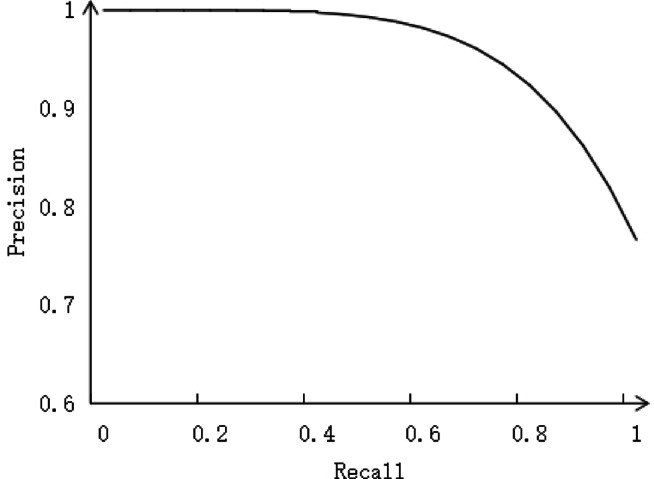
Precision-recall curve.

The Precision-Recall curve is shown in the figure below (Fig. 12).

**Fig. 13:**
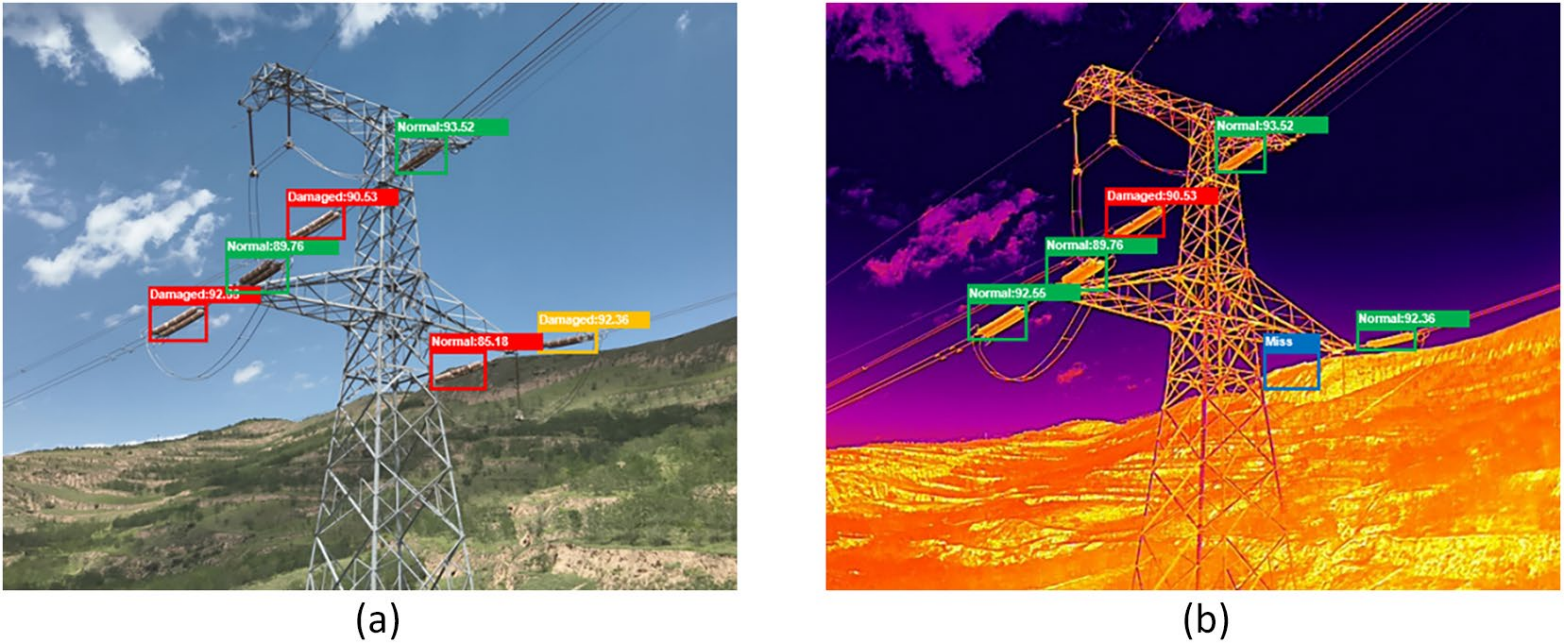
Case analysis of missing and false detections.

The following figure shows the missing detection and false detection cases of the model. In the visible light image on the left, the insulator marked by the orange bounding box is falsely detected as faulty, mainly because there are color differences between different insulators in the insulator string. The model failed to accurately identify this situation and mistook the insulator discs with different colors for missing ones. In the infrared image on the right, the insulator marked by the blue bounding box is missed, mainly affected by resolution and shooting angle. This insulator string has little difference from the environmental background, leading to the model’s failure to recognize it (Fig. 13).

## Discussion

To intuitively demonstrate the detection effect of the improved algorithm, this study compared the detection effects of the improved CWSP-YOLO model with those of RetinaNet, Faster RCNN, RT-DETR, YOLOv5, YOLOv8, YOLOv9, YOLOv11 and YOLOv13 model. Using mean Average Precision (mAP), Precision (P), Recall (R), and frame rate as evaluation metrics, a comparative analysis was conducted between the above four mainstream models and the improved YOLOv11 model on the self-built dataset. The experimental results are shown in Table 4.

**Table 4:** Dataset comparison experiment.

MODEL	mAP50	mAP	*P* (%)	*R* (%)	FPS (f/s)
RetinaNet	73.45	50.26	82.36	70.64	10
Faster RCNN	74.45	54.32	83.47	70.13	13
RT-DETR	80.16	60.88	88.53	74.69	21
YOLOv5	62.43	41.19	71.78	60.37	14
YOLOv8	65.83	46.43	74.69	61.56	18
YOLOv9	73.66	55.23	78.15	66.58	16
YOLOv11	77.92	59.18	86.22	71.71	22
YOLOv13	82.15	59.98	90.18	79.78	25
ME-YOLO	79.32	58.45	91.07	77.52	20
BPP-YOLO	76.89	55.63	89.42	75.84	23
ACFI-YOLO	82.56	60.72	93.28	81.15	19
CWSP-YOLO	84.77	61.15	94.53	82.38	24

The data in Table 4 presents the detection performance and detection speed of each model. According to the results, RetinaNet can maintain good insulator defect recognition accuracy, but its high complexity makes its detection speed difficult to meet the needs of practical inspection. Similarly, although Faster RCNN improves the frame rate to a certain extent while enhancing detection accuracy, its speed still fails to satisfy practical production requirements. YOLOv5 maintains a relatively high detection speed, yet its insulator defect recognition accuracy decreases to a certain degree. The original version of YOLOv11 and YOLOv13 ,and ME, BPP, and ACFI-YOLO perform well in both detection accuracy and detection speed, but there are still room for optimization.

**Fig. 14:**
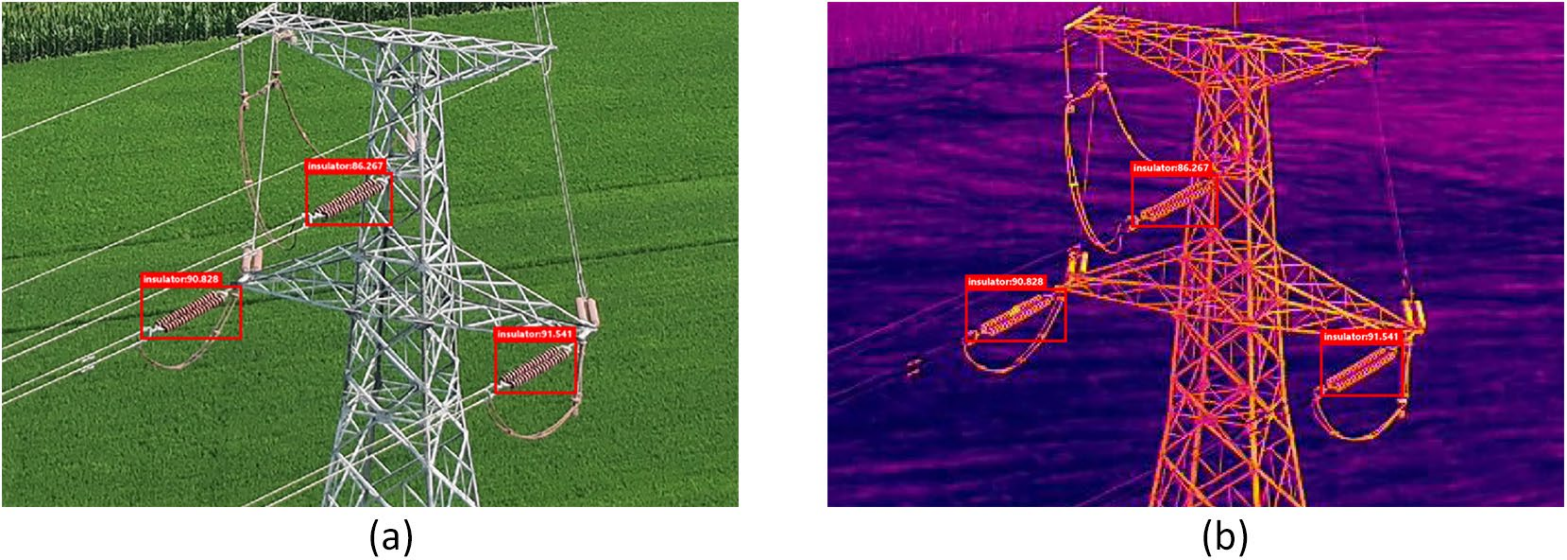
Visual comparison of insulator defect detection results.

The trained model was deployed on a UAV equipped with Jetson Orin Nano to test its inference capability on lightweight edge devices. Figure 14 shows the visualized comparison results of the detection in the comparative experiment, where the original YOLOv11 and CWSP-YOLO were selected for performance testing. In the figure, the “error” and “insulator” detection boxes correspond to fault locations and insulator categories, respectively, and the numbers marked in the upper left corner represent the predicted confidence. Analysis of Fig. 14a reveals that the original YOLOv11 model has issues such as missing detections of insulators and faults. From Fig. 14b, it can be observed that the CWSP-YOLO model accurately identifies all insulators in the figure, and the regions it frames better match the actual sizes of the insulators. Meanwhile, the comparison indicates that the improved model can achieve accurate localization and classification of multiple targets: it can identify the positions of insulators and accurately detect abnormal heating areas through multi-spectral fusion.

From the comprehensive experimental results, it can be seen that the optimized YOLOv11 model, while maintaining high detection efficiency, exhibits better overall performance in the key performance indicators—including mean Average Precision, Precision, Recall, and frame rate—for insulator defect recognition.

Meanwhile, we also compared the efficiency differences of the model on the RTX 4090 and Jetson Orin Nano. It can be seen that on edge computing devices, the algorithm’s processing efficiency can still meet the engineering requirements, which demonstrates the superiority of the algorithm.

**Table 5:** Dataset comparison experiment.

No.	Preprocess (ms)	Backbone (ms)	Neck (ms)	Head (ms)	NMS (ms)	Total (ms)	FPS (f/s)
RTX4090	1.5	15.8	12.5	4.8	7.07	41.67	24
Jetson Orin Nano	4.3	49.2	37.8	14.5	9.31	111.11	9

It should be further supplemented that the SE attention mechanism reduces the false detection rate of complex backgrounds through a channel-wise feature selection mechanism: first, it uses global average pooling to “squeeze” the features of each channel, capturing the overall distribution of insulators and backgrounds to break through the limitation of local perception; then, it uses fully connected layers to “excite” and learn weights, assigning high weights to defect-related effective channels and low weights to background-dominant channels; finally, it realizes the strengthening of defect features and suppression of background features through “re-calibration”, enabling the detection module to focus on valid information and fundamentally reduce the confusion between background and defect features. Wavelet Decomposition improves the detection sensitivity for small defects through a multi-scale detail separation and enhancement mechanism: it decomposes the image into low-frequency global contours and multi-scale high-frequency detail components; the features of small defects (such as tiny cracks and local contamination) are exactly concentrated in the high-frequency components, thus separated from the overall contours of insulators and complex backgrounds; meanwhile, it can specifically enhance the response intensity of high-frequency components and weaken the interference of low-frequency backgrounds, making the originally weak small defect features more prominent; finally, the detection model can accurately capture these amplified detail information, greatly improving the perception ability for small defects.

## Conclusions

Aiming at the challenge of insulator defect detection in complex scenarios during UAV power inspection, this paper proposes an improved model based on YOLOv11 with multimodal data fusion. By constructing a cross-modal collaborative perception mechanism and a mid-stage fusion strategy, it effectively achieves feature complementarity between visible light and infrared modalities, significantly enhancing the representational capability of defect regions. The introduced channel attention mechanism improves the model’s recognition accuracy of insulators under complex background interference by strengthening key feature channels. Furthermore, the PIoU v2 loss function effectively balances the gradient contributions of anchor boxes with different qualities, accelerating the model’s convergence process.

Experimental verification based on the self-built multimodal aerial insulator dataset shows that this method outperforms baseline models (YOLOv11) in key indicators, including Average Precision (84.77%), Precision (94.53%), and Recall (82.38%), while maintaining a real-time detection speed of 24 FPS. The proposed method not only effectively solves the false detection and missing detection issues of traditional detection methods in complex scenarios but also provides an intelligent detection solution with high precision and strong robustness for UAV power inspection projects.

## Data Availability

The raw data and original datasets supporting the findings of this study cannot be made publicly available due to restrictions related to commercial confidentiality and power grid security. The commercial confidentiality restrictions stem from the inclusion of proprietary technical parameters and operational data of the collaborating power enterprise, which are protected by non-disclosure agreements. The power grid security restrictions arise because the datasets involve sensitive information about power grid topology and load operation, the disclosure of which may pose risks to national energy infrastructure security.To ensure the reproducibility of the research, the processed summary datasets that support the key findings of this study are available from the corresponding author upon reasonable request. Details of commercially available materials used in the study (e.g., detection instruments, standard reagents) are listed in the Methods section.
